# Characterizing healthcare resource utilization in two rare diseases (Kleefstra syndrome and SLC6A1 epileptic encephalopathy) using multimodal real-world data

**DOI:** 10.1186/s13023-025-03879-x

**Published:** 2025-07-07

**Authors:** Caitlin A. Nichols, Ella Nysetvold, Mike Jackson, Ainslie Tisdale, Christine M. Cutillo, Shannon Rego, Ashley N. Cogell, Nelson D. Pace, Kristina Cotter

**Affiliations:** 1 AllStripes Research, San Francisco, CA USA; 2 National Center for Advancing Translational Sciences (NCATS), National Institutes of Health (NIH), Bethesda, MD USA

**Keywords:** Diagnosis, Healthcare resource utilization, Kleefstra syndrome, Observational study, Patient registry, Patient-reported outcomes, Real-world data, Real-world evidence, SLC6A1 epileptic encephalopathy, Survey

## Abstract

**Background:**

The cumulative economic burden of rare diseases surpasses that of common conditions, yet patterns of healthcare resource utilization (HRU) across rare diseases remain poorly characterized. This study leverages multimodal data collected during clinical care and through surveys to provide an in-depth evaluation of HRU across the disease journey of individuals with rare genetic diseases. Individuals with a confirmed diagnosis of Kleefstra syndrome (KS; n = 40) or SLC6A1 epileptic encephalopathy (SLC6A1; n = 30) were recruited. Structured and unstructured data were abstracted from participants’ medical records. Encounters per person-year of follow-up were calculated and compared pre- and post-diagnosis. Parents/guardians completed surveys assessing the impact of the participant’s diagnosis on their care.

**Results:**

Records were available for a median of 6.4 years of follow-up from 268 unique healthcare facilities (median per patient = 4.5 facilities). Numbers of healthcare encounters were not significantly different 1 year pre- and post-diagnosis for either condition; however, the proportion of specialty encounters pre- and post-diagnosis varied significantly. Genetics encounters decreased for both conditions post-diagnosis. Cardiology, sleep medicine, and radiology encounters increased in KS post-diagnosis; conversely, audiology encounters decreased in KS post-diagnosis, and radiology encounters decreased in SLC6A1 post-diagnosis. Among specialty encounter types assessed, general practitioner (e.g. primary care, including pediatrics) encounters were the most common type for KS participants and the second-most common for SLC6A1 participants (after neurology encounters) both 1 year pre- and post-diagnosis. The number of both echocardiograms and electrocardiograms (ECG) significantly increased in KS 1 year post-diagnosis. 68% of survey respondents indicated that the participant’s care changed post-diagnosis.

**Conclusions:**

Though there was no significant difference in the number of encounters pre- and post-diagnosis, significant changes in types of HRU suggest that diagnosis leads to more appropriate care and treatment. Advocacy organizations, researchers, drug developers, payors, and policymakers should consider the value of an early diagnosis to improve long-term outcomes and quality of life for patients and invest in measures that will shorten the time to diagnosis accordingly.

**Supplementary Information:**

The online version contains supplementary material available at 10.1186/s13023-025-03879-x.

## Background

In the United States, a rare disease is defined as a condition affecting fewer than 200,000 people [1]. Although uncommon individually, collectively rare diseases are prevalent: in the United States alone, rare diseases affect about 30 million or 1 in 10 Americans [2]. In addition to the burdens faced by individual patients and caregivers, rare diseases in aggregate have a substantial impact on the healthcare system [3]. Recent studies have highlighted the economic burden of rare diseases and the significantly higher healthcare utilization and costs for individuals with rare diseases compared to those with more common conditions [4, 5].

In addition to differences in healthcare resource utilization (HRU) between conditions, HRU can also vary by stage in the disease journey [6]. Diagnosis marks an important milestone after which patterns of HRU may change, and previous studies have examined these changes for various health conditions. For example, in the non-rare diseases endometriosis, Alzheimer’s disease, and celiac disease, studies have shown increased HRU and/or cost of care over various time periods post-diagnosis [7–9]. However, a study of individuals with Dravet syndrome, a rare pediatric epilepsy, showed an overall decreased HRU after diagnosis [10]. The results of these studies are dependent on multiple factors, including the specific clinical course of disease, treatment availability, and disease severity, among others. Further elucidating the relationship between HRU and diagnosis can inform patients, caregivers, and providers on care trajectories and cost changes after diagnosis. Additionally, this understanding can provide insight into the overall financial burden of different conditions on the healthcare system [9], improve understanding of treatment effectiveness [11, 12], and aid in early identification and confirmation of a diagnosis.

Despite the demonstrated utility of HRU data to both patients and providers, HRU remains poorly characterized in most rare diseases, especially for those that lack disease-specific identifiers, such as International Classification of Diseases (ICD) or Systematized Nomenclature of Medical – Clinical Terms (SNOMED CT) codes. More than half of all rare diseases do not map to an ICD-10 code and many do not have specific SNOMED CT codes. Additionally, interoperability between coding systems is sometimes flawed, making it difficult to obtain information on these conditions from traditional databases [13–15].

In order to address this knowledge gap of HRU in rare diseases, we explored the relationship between HRU and diagnosis for individuals with 2 rare, pediatric-onset conditions: Kleefstra syndrome (KS; OMIM 610253) and SLC6A1 epileptic encephalopathy (SLC6A1; OMIM 616421). These rare, autosomal dominant disorders both affect young children and can cause developmental delays and intellectual disability, as well as other multisystemic manifestations in KS and seizures in SLC6A1. KS is caused by loss-of-function pathogenic variants in the *EHMT1* gene or subtelomeric deletions at chromosome 9q34 overlapping all or part of *EHMT1* [16, 17]. EHMT1 encodes euchromatin histone methyltransferase 1, which methylates the lysine-9 position of histone H3 [18]. Deficiency of this epigenetic factor has been shown to impair normal neuronal activity during development [19], and individuals with KS exhibit altered DNA methylation patterns relative to control individuals [16]. SLC6A1 is caused by pathogenic variants in the *SLC6A1* gene, which encodes the voltage-dependent c-aminobutyric acid (GABA) transporter 1 (GAT-1) protein, one of the major GABA transporters of the human central nervous system [19]. SLC6A1 is primarily expressed in the brain, specifically in GABAergic neurons and astrocytes [20]. Pathogenic variants in SLC6A1 cause loss of GAT-1 function and reduced GABA reuptake from the synaptic cleft [19]. The incidence of KS is not well defined; prevalence estimates range from 1 in 25,000 to 1 in 35,000 individuals, but the actual prevalence may be higher due to underdiagnosis [21]. SLC6A1-related disorders are estimated to occur in 2.7 per 100,000 births [22], and fewer than 500 individuals have been reported worldwide [23]. To date, neither condition has a disease-modifying treatment available [21, 24] nor a specific ICD-10 code, making studies of HRU that rely solely on claims or EHR databases challenging. Here, we leverage multimodal real-world data collected during clinical care (e.g., structured and unstructured EHR data, digitized paper medical records, long-form clinical notes, etc.) and through surveying participants to evaluate individuals’ HRU across the disease journey, specifically focusing on how HRU changes following diagnosis.

## Methods

AllStripes Research is a medical data company specializing in rare diseases. Patients and legally authorized representatives who join an AllStripes research program can access their digitized and organized medical records via a secure platform, through which they can provide consent for data in their records to be used in research studies. Individuals living with KS or SLC6A1 in the United States, United Kingdom, and Canada were recruited to join the AllStripes platform via digital marketing. A tailored approach was implemented to recruit individuals with KS or SLC6A1 by working closely with AllStripes Ambassadors (caregivers who choose to participate in individual advocacy and share their experiences with KS or SLC6A1; no other incentives were provided) and patient advocacy groups (iDefine and SLC6A1 Connect). Recruitment tools included virtual flyers, webinars co-hosted with the patient advocacy groups, and AllStripes Ambassador stories shared on the AllStripes website. These recruitment tools were shared on social media and within patient advocacy groups, and AllStripes Ambassadors for KS or SLC6A1 were able to share them within private KS or SLC6A1 community groups if they chose to do so. Given limited data availability on HRU patterns in rare diseases, multiple indications were selected for this study. Complex rare indications with limited or no previously published HRU data were considered. KS and SLC6A1 both met these criteria and both programs had strong support from the respective communities.

Parents or legal guardians of individuals with KS or SLC6A1 were directed to a disease-specific recruitment page [25, 26], where they were able to (1) sign a HIPAA release (United States/Canada) or Subject Access Request (United Kingdom) form authorizing AllStripes to request the participants’ medical records on their behalf; (2) provide a list of hospitals and clinics where the individual received care, from which AllStripes would request participant medical records; and (3) sign an eConsent form allowing for participant recontact, survey data collection, and the use of de-identified data from medical records in minimal-risk research. After signing authorization forms, parents/guardians were prompted to provide basic participant demographics, including biological sex, age, birth date, race, ethnicity, and country of residence. Parents/guardians of consented participants were also given the opportunity to complete a survey on behalf of the participant about how the individual’s diagnosis impacted care, treatment opportunities, and overall quality of life. Medical records were used as a secondary source to determine participant biological sex when that information was missing from incomplete enrollment surveys.

The study protocol and all study materials were approved by WCG IRB. This study consisted of the evaluation of data from two main sources: participant medical records and a survey about the impact of receiving a diagnosis.

### Medical record–based evaluation

Participants’ medical records (including structured and unstructured electronic health records and paper records) were requested from all reported healthcare facilities in the United States, United Kingdom, and Canada, including academic centers of excellence and community healthcare settings. Five email or phone requests for medical records were made to each listed facility, at least once per week. Additional requests were made if the facility engaged on or before the fifth request; however, if after five requests the facility failed to respond or indicated that they did not have the requested records, requesting activity to that facility would cease. In order to address potential data gaps, medical records were also requested from additional healthcare facilities as appropriate. Parents/guardians were contacted to identify additional clinicians/healthcare facilities if gaps in time, clinician specialty, or document type were identified in a patient’s chart. All medical records and other identifiable information were securely stored on the AllStripes platform.

Once received by AllStripes, medical records were digitized and organized by clinical operations associates, including classification according to clinical specialty and document type (Additional File 1), and made available to participants on the secure AllStripes platform. Operations associates involved in the processing of medical records were provided with standard guidance documents and completed a training program and practical assessment. Gaps identified during the assessment were addressed through individualized, practical coaching. Medical record scenarios not addressed in relevant guidance documents were escalated to an operations team lead. Medical record processing was assessed for quality by a second member of the operations staff trained in quality control processes.

Clinical abstractors consisted of registered nurses, nurse practitioners, genetic counselors, or healthcare professionals with 5 + years of clinical research management or abstraction experience. All abstractors completed a foundational training course and practical skills assessment. Gaps identified during the assessment were addressed through individualized, practical coaching.

For this study, abstractors were provided with study- and variable-specific guidance documents and received condition-, study-, and variable-specific training. Abstraction scenarios not addressed in relevant guidance documents were escalated to an abstraction team lead or research team lead. Abstracted data were evaluated in a two-step quality control process involving primary review by an abstraction team lead and secondary review by a research team member with clinical and/or biomedical sciences training.

Specific free-text data points were captured by clinical abstractors from medical records available to AllStripes prior to 11/2/21. Variables abstracted for this study include the following: diagnosis date; diagnosing clinician specialty; symptoms and corresponding onset date; surgery/procedure type, date, and indication; emergency department (ED) and inpatient visit date, duration, and indication; medications prescribed and corresponding start and stop dates; electroencephalogram (EEG), electrocardiogram (ECG), and echocardiogram dates; and any documented therapies. An electronic audit trail was maintained for quality assurance, and captured data was additionally reviewed for quality control.

The number and type of medical documents (determined using a compiled record classification dataset indicating specialty and document type for each record) was used to calculate the number and type of healthcare encounters per participant. Only documents corresponding to a visit (e.g., notes, testing reports) were considered “encounters”; certain documents and records, as determined by AllStripes’ proprietary quality control processes, were excluded to improve accuracy and reduce redundancy in calculating participant encounters. To account for potential duplication of medical documents within a participant’s chart, documents from the same date of service and medical specialty were considered a single unique encounter. Eligible documents received by AllStripes prior to 12/1/21 were included in analyses based on medical record–classification data.

### Impact of diagnosis survey

Informed by previous studies [4], we developed a survey about how the participant’s diagnosis impacted care, treatment opportunities, and overall quality of life. The survey was piloted with one rare disease patient and one caregiver of a child living with a rare disease prior to its use in this study. No validated instruments were embedded in this survey.

The survey consisted of a maximum of 28 questions. Six core questions were shown to all respondents. The first five core questions asked whether the participant’s medical care, access to care/resources, medical costs, indirect costs, and quality of life have changed post-diagnosis. Based on responses to these core questions, respondents were asked additional questions related to the five core topics, with 22 additional questions possible. For example, if a respondent indicated that the cost of the participant’s medical care had changed since diagnosis, they would also be asked about the magnitude and direction of the change and what contributed to the change. The final core question asked if there was anything else the respondent wanted to share about the impacts of the patient receiving a diagnosis of KS or SLC6A1.

### Inclusion/exclusion criteria

In order for a participant to be included in the medical records-based portion of the study, individuals’ collected records were required to contain at least 1 neurology, genetics, or psychology/psychiatry clinician note (KS) or at least 1 neurology or genetics note (SLC6A1) within the previous year. Only participants with medical records from both the pre- and post-diagnosis time periods were eligible for study inclusion. Additionally, only individuals with a genetically confirmed diagnosis of KS or SLC6A1 were screened into the study. Genetically confirmed diagnoses were defined as follows: pathogenic or likely pathogenic variants (or equivalent classifications such as “positive”, “causative”, “likely causative”, etc.) in EHMT1 or SLC6A1, respectively. One KS participant with a variant of uncertain significance in EHMT1 was included because their geneticist believed the variant to be diagnostic as per clinical documentation and the variant has since been reclassified by the testing laboratory as likely pathogenic. Throughout the study, the diagnosis date was defined as the date the diagnosis was genetically confirmed.

Parents/guardians of all consented participants were invited to complete the survey about the impact of diagnosis on behalf of the participant; however, only responses for participants with a genetically confirmed diagnosis of KS or SLC6A1 were included in this study.

### Confidentiality

To ensure confidentiality, participants were assigned a unique study ID number. The key matching the study number with participant information was securely maintained by AllStripes Research. Only trained, essential personnel had access to identifiable information.

De-identified, patient-level study data (from medical record abstraction and surveys) were securely stored on the AllStripes platform separately from identifiable data. Personal identifiers were not included as a part of study data. All data underwent quality review to ensure personally identifiable information was not included in study data.

### Statistical analysis

All statistical analysis was conducted in R (version 4.1.0). Negative binomial regressions were performed using the glm.nb function from the MASS package to calculate rate ratios (RR) and 95% confidence intervals to determine whether there was a significant difference between the number of healthcare encounters relative to diagnosis (pre-diagnosis vs. post-diagnosis). Comparisons of 3-month (90-days) or 1-year time windows pre- and post-diagnosis were evaluated to assess the most immediate effects of diagnosis on HRU while aiming to minimize potential confounding that could occur with longer time windows evaluated. Several combinations of healthcare encounter types were evaluated using these time windows. Age at diagnosis, age at symptom onset, and length of hospitalization were calculated based on dates abstracted from participant medical records. All dates missing day or month granularity were imputed, when necessary for analysis, to the 15th day of the month or the 7th month of the year, respectively. Person-time of follow-up was calculated as the difference between the earliest and most recent dates of available medical documents. Number of encounters per person-year of follow-up was determined by dividing the number of encounters of each specialty type by observed person-time for each individual. To investigate the null hypothesis that no changes in HRU occurred post-diagnosis compared to pre-diagnosis, chi-squared and paired t-test calculations were performed on medical record classification data to compare specialty encounters for the time periods 1 year pre-diagnosis vs. 1 year post-diagnosis. Paired t-tests were also performed on abstracted medication, hospitalization, EEG, echocardiogram, and ECG data to compare utilization 1 year pre-diagnosis vs. 1 year post-diagnosis. The null hypothesis was rejected if the aforementioned tests produced a *p*-value < 0.05.

## Results

### Participant recruitment

Ninety-seven individuals with KS and 50 individuals with SLC6A1 were recruited to the AllStripes Research platform; of these, 40 KS and 30 SLC6A1 participants were eligible for inclusion in this study based on medical record completeness and diagnosis verification requirements (see Methods).

### Basic demographics and clinical characteristics

**Fig. 1:**
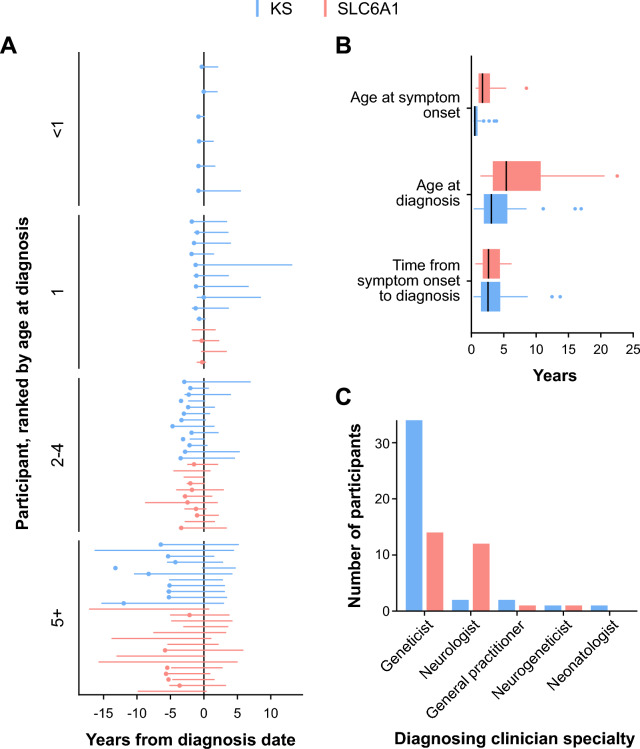
Diagnostic odyssey of participants living with Kleefstra syndrome (KS) and SLC6A1. **A** Symptomatic onset (dots) and length of medical record follow-up (red, blue lines) for each individual relative to their diagnosis date (year 0), with individuals grouped by age at diagnosis. **B** Age at symptom onset, age at diagnosis, and time from symptom onset to diagnosis by condition. Individuals with date of symptom onset recorded: KS, n = 38; SLC6A1, n = 16. Median time from symptomatic onset to diagnosis: KS = 2.20 years; SLC6A1 = 2.30 years. **C** Number of individuals diagnosed by various clinical specialties

Seventy participants were included in the medical records-based portion of the study (KS = 40, SLC6A1 = 30). Across both conditions, individuals had an average of 162.0 available medical documents (median = 119.5) and an average of 7.1 years of follow-up (median = 6.4). Most participants were female (44 of 70, 63%) and diagnosed at less than 6 years of age (Table 1, Fig. 1a). Almost all participants were located in the United States (68 of 70, 97%).

**Table 1:** Demographics and medical record statistics for individuals participating in medical-records–based portion of the study

	KS (N = 40)	SLC6A1 (N = 30)	Total (N = 70)
*Age*			
Mean (SD)	7.20 (4.50)	9.33 (6.12)	8.11 (5.32)
Median [Min, Max]	6.50 [1.00, 21.0]	8.00 [1.00, 25.0]	7.00 [1.00, 25.0]
*Biological sex*			
Female	24 (60.0%)	20 (66.7%)	44 (62.9%)
Male	16 (40.0%)	10 (33.3%)	26 (37.1%)
*Race*			
More than one race	2 (5.0%)	4 (13.3%)	6 (8.6%)
White	28 (70.0%)	23 (76.7%)	51 (72.9%)
Asian	0 (0%)	1 (3.3%)	1 (1.4%)
Black or African American	0 (0%)	1 (3.3%)	1 (1.4%)
Unknown	10 (25.0%)	1 (3.3%)	11 (15.7%)
*Ethnicity*			
Hispanic or Latino	2 (5.0%)	4 (13.3%)	6 (8.6%)
Not Hispanic or Latino	27 (67.5%)	25 (83.3%)	52 (74.3%)
Prefer Not to Say	1 (2.5%)	0 (0%)	1 (1.4%)
Unknown	10 (25.0%)	1 (3.3%)	11 (15.7%)
*Total number of documents*			
Mean (SD)	149 (118)	179 (196)	162 (156)
Median [Min, Max]	120 [24.0, 599]	118 [47.0, 1070]	120 [24.0, 1070]
*Years of records*			
Mean (SD)	6.63 (4.48)	7.69 (4.61)	7.09 (4.53)
Median [Min, Max]	5.52 [1.00, 20.8]	6.92 [1.38, 20.8]	6.41 [1.00, 20.8]

KS; Kleefstra syndrome

Individuals living with KS were diagnosed at a median of 2.7 years of age and had symptom onset at 0.2 years of age; individuals with SLC6A1 were diagnosed at 5.0 years and had symptom onset at 1.1 years (Fig. 1b). The most common symptoms present before diagnosis were hypotonia (32/40, 80%) and dysmorphic facial features (23/40, 57%) for KS and seizures (19/30, 63.3%) and hypotonia (15/30, 50%) for SLC6A1. Median time from symptom onset to diagnosis was similar for both cohorts (KS = 2.2 years, SLC6A1 = 2.3 years; Fig. 1b), and the majority of participants in both cohorts were diagnosed by a geneticist as recorded in a first-hand genetics clinical note or historical documentation from another care provider (KS: 34/40, SLC6A1: 14/30, Fig. 1c).

### Overall HRU

**Fig. 2:**
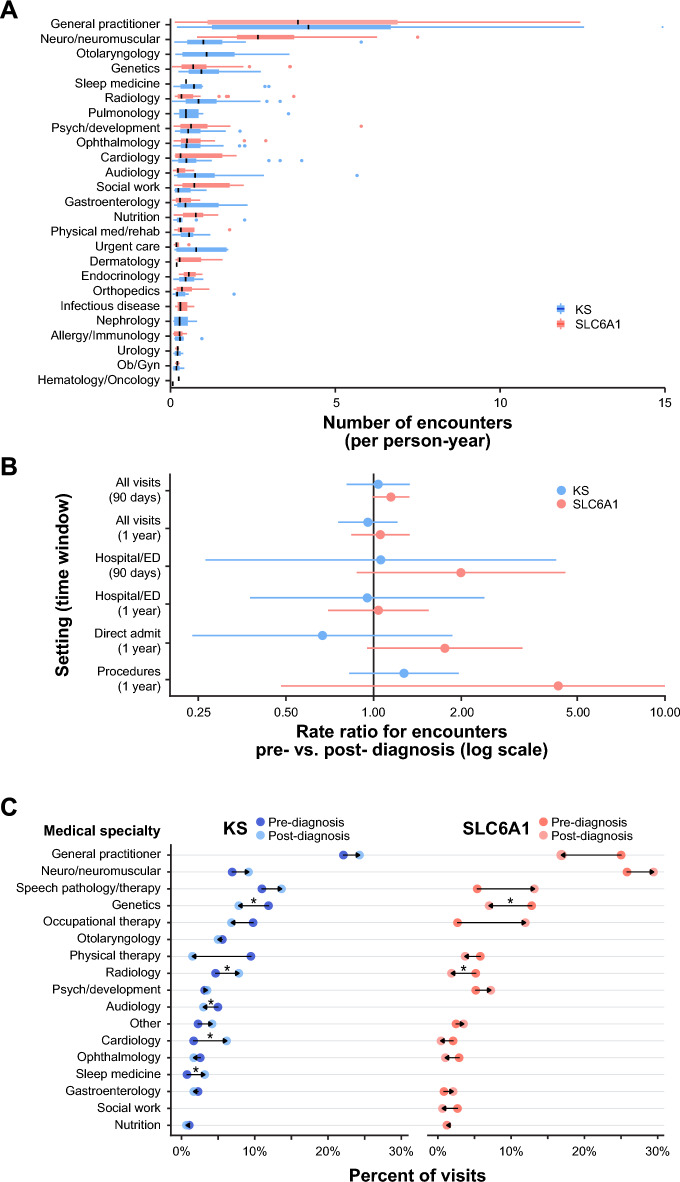
Quantity and types of HRU for participants with Kleefstra syndrome (KS) and SLC6A1 pre- and post-diagnosis. **A**. Number of encounters (based on medical record specialty and date) per person-year of data. [Note: One outlier for general practitioner encounters exceeded 15 per year and is not represented in Panel A.] **B**. Negative binomial regression analysis of pre- and post-diagnosis rate ratios (RR) for encounters (dots = RR, lines = 95% CI). 90-days (d) or 1-year (y) time windows pre- and post-diagnosis were used to minimize potential confounding due to changes over time. Analyses included all encounters, emergency department (ED)/hospital encounters only, direct hospital admissions only, or procedures only. **C**. Percent of encounters attributed to various specialties 1 year pre- and post-diagnosis. Overall specialty composition varied significantly pre- and post-diagnosis for both conditions (*p* < 0.05). Paired t-tests were performed for each encounter specialty 1 year pre- and post-diagnosis; * indicates a significant difference (*p* < 0.05)

General practitioner encounters were the most common visit type across the study follow-up period for both conditions (Fig. 2a). Across available records, most participants with KS had at least 1 recorded surgery or procedure (68%), and most with SLC6A1 had at least 1 direct hospital admission (63%) (Additional File 2). Ninety percent of SLC6A1 direct admissions (34/38) were indicated for seizures or EEG monitoring. Forty-three percent of participants in the study had at least 1 emergency department (ED) visit, and 27% had an ED visit that led to hospital admission (Additional File 2). For both conditions, most participants were prescribed at least 1 medication and had a record of at least 1 therapy (e.g., physical therapy; Additional File 2). Polyethylene glycol, a laxative, was the most commonly prescribed medication for KS (13/40, 33%), and levetiracetam, an anticonvulsant, was the most commonly prescribed medication for SLC6A1 (17/30, 57%).

### HRU relative to diagnosis

The following encounter categories were analyzed: all encounters (based on medical record–classification data), all ED/hospital encounters only, direct hospital admissions only, and procedures only. Encounter rates were not significantly different pre- vs. post-diagnosis for any encounter type for either condition (*p* > 0.05) (Fig. 2b). The rate of medication prescription for medications prescribed 1 year pre- and post-diagnosis did not significantly differ for either condition (paired t-test, KS: *p* = 0.093, SLC6A1: *p* = 0.426).

As the quantity of healthcare encounters did not vary significantly pre- vs. post-diagnosis, we were interested in understanding whether encounter types changed with diagnosis. A chi-square test comparing the overall composition of encounters pre- and post-diagnosis showed that the percent of encounters attributed to various specialties 1 year pre- and post-diagnosis varied significantly (KS: *p* < 0.001, SLC6A1: *p* < 0.001). We further performed paired t-tests comparing the rate of each specialist encounter type 1 year pre-diagnosis and 1 year post-diagnosis (Fig. 2c). We observed a significant decrease in genetic specialty encounters after diagnosis for both conditions (KS: *p* < 0.001, SLC6A1: *p* = 0.027). We also observed a significant increase in cardiology, sleep medicine, and radiology encounters for KS participants (*p* = 0.001, *p* = 0.025, p = 0.037). Audiology encounters decreased post-diagnosis for KS participants (*p *= 0.037), and radiology encounters decreased post-diagnosis for SLC6A1 participants (*p* < 0.001). Among the encounter types assessed, general practitioner encounters were the most common type for KS participants and the second-most common for SLC6A1 participants (after neurology encounters) both 1 year pre- and post-diagnosis (pre- vs. post-diagnosis: KS, *p* = 0.977; SLC6A1, *p* = 0.077).

**Fig. 3:**
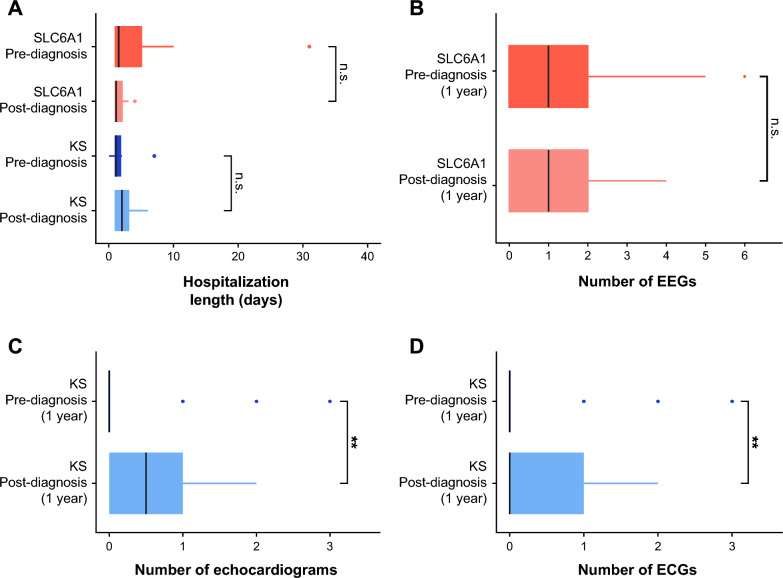
Hospitalization length and disease-related testing pre- and post-diagnosis. **A** Hospital admission length 1 year pre-/post-diagnosis for Kleefstra syndrome (KS) and SLC6A1 participants. Length of admission in days did not differ significantly pre- and post-diagnosis for individuals with KS (median: pre-diagnosis = 1 day (d), post-diagnosis = 2 d, two-sample t-test: *p* = 0.619, n.s. = not significant) or SLC6A1 (median: pre-diagnosis = 1.5 d, post-diagnosis = 1 d, *p* = 0.172, n.s. = not significant). **B** Number of electroencephalograms (EEG) for SLC6A1 participants 1 year pre-/post-diagnosis (paired t-test, p = 0.713, n.s. = not significant). **C** Number of echocardiograms for KS participants 1 year pre-/post-diagnosis (paired t-test, **p = 0.002). **D** Number of electrocardiograms (ECG) for KS participants 1 year pre-/post-diagnosis (paired t-test, **p = 0.010)

We also examined potential changes in duration of hospitalization or frequency of disease-related testing relative to diagnosis using data abstracted from participant medical records. Hospitalization duration did not significantly change for either condition pre- vs. post-diagnosis (KS: *p* = 0.619, SLC6A1: *p* = 0.172; Fig. 3a). We also assessed potential changes in EEG administration for individuals living with SLC6A1 before and after diagnosis, including EEGs performed in inpatient and outpatient settings and at home. The total number of EEGs was not significantly different 1 year pre- versus 1 year post-diagnosis (paired t-test, *p* = 0.713; Fig. 3b). Likewise, the number of seizure/EEG-related direct admissions in SLC6A1 did not differ significantly 1 year pre- versus 1 year post-diagnosis (paired t-test, *p* = 0.787). We also examined changes in the number of cardiology-related tests for individuals with KS. The number of both echocardiograms and electrocardiograms (ECG) significantly increased 1 year post-diagnosis versus 1 year pre-diagnosis (paired t-test, echocardiogram: *p* = 0.002, ECG: *p* = 0.010; Fig. 3c and 3 d).

### Impact of diagnosis survey

Parents/guardians of 30 KS and SLC6A1 participants started a survey on behalf of the participant about how receiving a diagnosis impacted the individual’s medical care, access to different types of care, and overall quality of life (n: KS = 8, SLC6A1 = 22). Two potential respondents who started the survey did not answer any survey questions (n: KS = 1, SLC6A1 = 1). All of the remaining 28 respondents answered all questions that they were shown based on their answers to the six core questions (see Methods; completion rate = 100%). However, completed surveys from three respondents were excluded from this study because the participants’ diagnoses could not be genetically confirmed. The final survey dataset consisted of responses from parents/guardians of 25 KS and SLC6A1 participants (n: KS = 6, SLC6A1 = 19; completion rate = 100%). Twelve of the 25 respondents (KS = 1, SLC6A1 = 11) were guardians of participants included in the medical records-based portion of the study (Additional File 3).

Most respondents described medical care changes as a result of the diagnosis (17/25, 68%), and most of these described the changes as major or moderate (10/17, 59%). Of those who noted a change to medical care, the most common change was to the number or type of visits to medical specialists (11/17, 65%). Respondents indicating a change in medical care post-diagnosis also described an increase in the number or type of clinical, imaging, or laboratory tests (10/17, 59%); an increase in the number or type of therapies the participant attended (8/17, 47%); and changes to treatments such as medications and diets (9/17, 53%).

Most respondents did not note a change in either direct (13/25, 52%) or indirect costs of managing the participant’s condition (12/25, 48%) post-diagnosis. Although we did not ask directly about the impact of a diagnosis on health insurance coverage, respondents frequently mentioned it in free-text survey responses. In some cases, the impact on insurance was positive, primarily because the diagnosis allowed the participant to qualify for public health insurance (Medicaid; n = 6/25, 24%). However, some respondents commented that insurance still did not cover services they viewed as necessary (5/25, 20%).

**Fig. 4:**
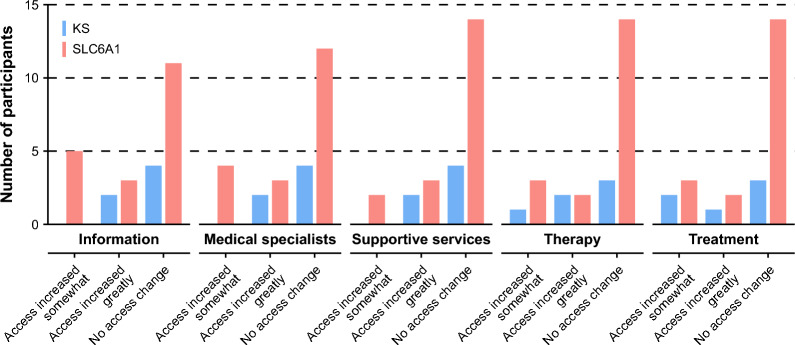
Change in access to care relative to diagnosis as indicated via impact of diagnosis survey. KS = Kleefstra syndrome

Respondents also described concrete benefits of diagnosis in terms of increased access to information, care, and/or support (13/25, 52%), with the most common increase being in access to information to manage the participant’s care (10/25, 40%; Fig. 4). Parent/guardian comments also supported this finding. One respondent wrote “Simply knowing what [the participant’s] diagnosis was provided direction and a wealth of information that would not have been looked at without the diagnosis” (Additional File 4, Quote 4.2). Another noted that “Doctors began believing that I needed the resources I kept asking to receive for my [child]” after the participant received a diagnosis of SLC6A1 (Additional File 4, Quote 2.1).

However, the majority of respondents (18/25, 72%) also indicated that the diagnosis did not improve the participant’s quality of life, citing ongoing challenges including “constant roadblocks” and having “no idea where life will lead” (Additional File 4, Quote 6.1). Only one parent/guardian indicated that quality of life had improved for their child, and two indicated worse quality of life after diagnosis.

## Discussion

Further characterizing the relationship between HRU and diagnosis can provide valuable insight into the burden of disease, inform patients and families about care trajectories and cost changes, and improve overall understanding of current treatment effectiveness. However, changes in HRU relative to diagnosis are not well understood for rare diseases, especially for rare diseases lacking condition-specific identifiers, such as ICD or SNOMED codes. This study examined HRU pre- and post-diagnosis for individuals with KS and SLC6A1, 2 rare, pediatric-onset conditions that do not yet have specific ICD-10 identifiers, using multimodal real-world data. The number of healthcare encounters remained approximately constant pre- vs. post-diagnosis, but the composition of specialties visited significantly changed after diagnosis for individuals with these conditions.

We found that the total amount of HRU did not differ significantly pre- vs. post-diagnosis for either condition. This finding may be related to the lack of disease-modifying therapies for both KS [21] and SLC6A1 [24]. In both conditions, the current standard of care focuses on symptom management (e.g., multisystemic symptom mitigation in KS [21], broad spectrum anti-seizure medication in SLC6A1 [24]). Because these symptomatic therapies do not address the genetic etiology of the disease, they likely do not decrease disease burden or HRU as effectively as disease-modifying treatments would. Alternatively, HRU rates in KS and SLC6A1 may decrease post-diagnosis due to improved management, but such decreases may be obscured by additional encounters to screen for or initiate preventative treatment for disease-associated symptoms of which caregivers were previously unaware—symptoms which may be better addressed by a disease-modifying therapy. In contrast, in cystic fibrosis (CF) and spinal muscular atrophy type 1 (SMA1), rare conditions that do have disease-modifying therapies, HRU metrics including risk of hospitalization or average number of hospitalizations have been shown to decrease in the months and years following treatment initiation [27, 28]. Given the tremendous burdens patients and families face as they manage a rare disease and the collective impact of rare diseases on the healthcare system, this finding underscores the importance of investing in the development of disease-modifying therapies. When accessible and affordable, such therapies offer the most promising path to decreasing the economic and psychosocial burdens experienced by individuals impacted by rare diseases like KS and SLC6A1 as well as the corresponding costs to society more broadly. The broader landscape of rare disease healthcare economics is of course complicated by the cost of therapies and individual payors’ willingness to reimburse for them; however, a discussion of these factors is outside the scope of this study. A study of healthcare costs in 12 individuals treated at a Czech hospital found a significant decrease in overall healthcare costs post-diagnosis in participants with Dravet syndrome (another rare pediatric epilepsy that lacks a disease-modifying therapy), including decreased costs for hospitalization and genetic testing [10]. Differences in these findings relative to our study may be driven by a number of factors, including differences in clinical practice and healthcare costs between countries, amount of information known about the respective conditions and how they are treated, underlying disease biology, and study sample size and design.

Although our analysis did not detect a significant change in the number of healthcare encounters pre- vs post-diagnosis, we did find that the composition of healthcare encounters changed significantly after diagnosis for individuals with both KS and SLC6A1. We hypothesize that these changes in care composition may be related to individuals receiving more condition-specific care after diagnosis. For example, rates of cardiology encounters and testing increased for participants living with KS post-diagnosis, consistent with cardiac issues known to be associated with KS. In SLC6A1, the rate of radiology encounters decreased post-diagnosis, perhaps indicating a decrease in imaging-related testing as patients move through the diagnostic period into condition management. Decreased genetics encounters for both conditions after diagnosis also supports the possibility of increased condition-specific care, as genetics teams may transition from actively managing a patient’s care and diagnostic odyssey to providing periodic follow-up post-diagnosis. Survey results support the possibility of qualitative changes in care after diagnosis: more than half of respondents indicated that care changed post-diagnosis (17/25, 68%) and that access to information, care, and/or support increased post-diagnosis (13/25, 52%). These results are similar to those obtained through other patient/caregiver surveys across various rare diseases in Australia [29] and Europe [30]. Together, these findings suggest that even without disease-modifying therapies, receiving a diagnosis still positively impacts treatment patterns and, potentially, care effectiveness. Additional research into the natural history of KS and SLC6A1 will assist with the development of clinical guidelines to improve patient care. Our findings on the value of the diagnosis are also consistent with other qualitative survey results from parents/guardians of individuals living with KS, including regarding the psychosocial impact of the additional knowledge and sense of community that can be derived as a result of diagnosis [31].

If diagnosis does in fact lead to more appropriate care for KS and SLC6A1, our findings also underscore the importance of earlier diagnosis for individuals living with these conditions. Investment in research and policies to help shorten the time to diagnosis may help patients access condition-specific care more quickly, ultimately improving long-term outcomes, including when disease-modifying drugs eventually enter the market. The results of this study suggest a potential approach to shortening time to diagnosis: improved education and awareness among pediatricians and family doctors. The high frequency of general practitioner visits both pre- and post-diagnosis illuminated in this study highlights the critical role these providers play in the care of patients living with KS and SLC6A1. Improved disease education and awareness among primary care providers could result in earlier recognition of warning signs, referrals to specialized care, and access to genetic testing, thus leading to earlier diagnosis and improved patient outcomes and quality of life. Future studies should further investigate the diagnostic odysseys of individuals living with KS and SLC6A1, including presenting symptoms, clinician referral patterns, and patterns of genetic testing that ultimately lead to genetic confirmation of disease. It is worth noting that both KS and SLC6A1 have patient advocacy groups that provide support to patients and families. This fact may contribute to the post-diagnosis increase in access to information and appropriate care that we identified in this study. Future studies should evaluate the generalizability of these findings to rare indications with less-established communities.

Several limitations to our study should be noted. Sample size for both the medical records-based and survey-based portions of the study was limited, with participants largely residing in the United States. Additionally, only individuals with a confirmed genetic diagnosis whose guardians read and understand English and had access to and were willing to participate in an online research program were included. The rare nature of both indications also required a tailored recruitment approach involving snowball sampling and close collaboration with patient advocacy groups and AllStripes ambassadors. This may have introduced potential selection bias for families who are well-connected in each disease community, and who may be more likely to seek information, medical care, or testing. Altogether, the HRU patterns of these individuals may differ from the general population of individuals living with KS or SLC6A1. Future studies should investigate the impact of socioeconomic factors and country of residence on HRU patterns in these and other rare indications.

The use of secondary data like medical records also introduces inherent challenges, such as the increased likelihood of missing or incomplete data variables compared to primary data collected as part of site-based studies and surveys. We addressed this limitation by obtaining lists of care providers and healthcare facilities directly from parents/guardians, who are best positioned to understand the patients’ entire medical journey across care settings. We also sourced additional facilities when potential gaps in care were identified. The challenge of potentially missing data impacts any study relying on retrospective chart review, including those at tertiary care centers. However, our ability to recontact parents/guardians to request additional information as well as our gathering records from diverse care settings, including community care centers, may provide a more nuanced picture of healthcare utilization than could be obtained through records from a single center of excellence. Finally, our method of identifying unique encounters from medical record classification data (limiting to a maximum of 1 encounter per specialty type per day) tends to underestimate rather than overestimate participants’ HRU; that said, we do not suspect differential underestimation pre-/post-diagnosis as this potential underestimation is anticipated to affect both time periods similarly.

There are several possible confounders that may have impacted HRU patterns. For example, the presence of additional conditions not evaluated by this study could have influenced clinical care; e.g., secondary findings identified during diagnostic genetic testing may have prompted additional clinical visits. The impact of secondary diagnoses and secondary genetic findings on HRU patterns are of interest for future study. Further, as additional data is published on the natural histories of KS and SLC6A1, it will be of interest to investigate whether the changes in specialty visit composition identified in this study could also be explained in part by symptomatic development or progression appropriate to the patient’s age or disease severity.

Another limitation to our study is the reliance on parents or guardians to respond to the survey on behalf of their children with KS or SLC6A1. While this approach introduces the possibility of parental perspectives influencing the responses, it is important to recognize that parents and other caregivers often serve as primary observers of their children’s experiences, thereby offering valuable critical insight into the overall impact of the diagnosis on both the child and the family unit.

## Conclusions

By consenting participants to research directly through a virtual, study site–agnostic model, we were able to characterize the HRU of individuals living with KS and SLC6A1, 2 conditions for which studies using traditional claims or EHR databases may prove challenging. Our use of multimodal data, including data collected during routine clinical care and through surveys, enabled us to marry data on HRU with the context found in medical notes and participants’ lived experiences. This study determined that while the quantity of participants’ HRU did not change after they were diagnosed with KS or SLC6A1, healthcare encounter composition did shift significantly following diagnosis. This result suggests that diagnosis leads to more appropriate or targeted care in these conditions. Advocacy organizations, researchers, drug developers, payors, and policymakers should consider the value of an early diagnosis to improve long-term outcomes and quality of life for patients and invest in measures that will shorten the time to diagnosis accordingly. However, the most promising path to decreasing the burden of these rare diseases on patients, families, communities, and the healthcare system remains the development of disease modifying therapies. Further research into the pathophysiology and natural history of KS and SLC6A1 and the journeys of individuals and families living with these conditions will be critical to the development and ultimate approval of such therapies.

## Supplementary Information

Additional file1

Additional file2

Additional file3

Additional file4

## Data Availability

The datasets generated during the current study are not publicly available due to them containing information that could compromise research participant privacy/consent. However, they may be available through an application process managed by PicnicHealth (PicnicHealth acquired AllStripes in 2023) and limited to researchers working with recognized academic or research organizations and who agree to protect the privacy of participants. In order to decrease the risk of participant reidentification, certain information may be redacted from any data shared, and dates may be shifted and/or provided with decreased granularity. Requests for data access may be directed to PicnicHealth.
