# Supplementary material for: Characterizing healthcare resource utilization in two rare diseases (Kleefstra syndrome and SLC6A1 epileptic encephalopathy) using multimodal real-world data
Source: Orphanet J Rare Dis. 2025 Jul 7;20:344. doi: 10.1186/s13023-025-03879-x (PMC12232648; doi:10.1186/s13023-025-03879-x)
Supplement: Supplementary file 1 — Additional file1 [file 13023_2025_3879_MOESM1_ESM.docx]

Additional File 1: Medical specialties and corresponding document types included in the medical records–based portion of the study.

**Medical specialty
 *Document types***

Allergy/Immunology
*Notes
Testing*

Audiology
*Audiogram
Notes*

Cardiology
*ECG
Echocardiogram
Notes*

Dermatology
*Notes*

Endocrinology
*Notes*

Gastroenterology
*Notes*

General practitioner
*Notes*

Genetics
*Notes
Testing*

Hematology/Oncology
*Notes*

Infectious disease
*Notes*

Nephrology
*Notes*

Neuro/neuromuscular
*EEG
Movement disorder notes
NCS/EMG study
Notes*

Nutrition
*Notes*

OB/GYN
*Birth notes
Notes*

Occupational therapy
*Notes*

Ophthalmology
*Notes
Testing*

Orthopedics
*Notes*

Otolaryngology
*Notes*

Palliative care
*Notes*

Physical medicine / rehabilitation
*Notes*

Psych / development
*Child life
Notes*

Pulmonology
*Notes
Respiratory therapy
Testing*

Radiology
*CT scan
Interventional radiology
PET scan
Ultrasound
X-ray*

Sleep Medicine
*Notes
Polysomnography*

Social work
*Notes*

Speech pathology / therapy
*Notes*

Urgent care
*Notes*

Urology
*Notes*
