# Supplementary material for: Characterizing healthcare resource utilization in two rare diseases (Kleefstra syndrome and SLC6A1 epileptic encephalopathy) using multimodal real-world data
Source: Orphanet J Rare Dis. 2025 Jul 7;20:344. doi: 10.1186/s13023-025-03879-x (PMC12232648; doi:10.1186/s13023-025-03879-x)
Supplement: Supplementary file 2 — Additional file2 [file 13023_2025_3879_MOESM2_ESM.docx]

**Additional File 2. Percent of participants with various types of HRU based on medical record review.** KS = Kleefstra syndrome, ED = emergency department.

|  | **KS**  **(N=40)** | **SLC6A1**  **(N=30)** | **Overall**  **(N=70)** |
| --- | --- | --- | --- |
| **Inpatient (direct admit)** | 14 (35.0%) | 19 (63.3%) | 33 (47.1%) |
| **Inpatient (from ED)** | 11 (27.5%) | 8 (26.7%) | 19 (27.1%) |
| **Emergency Department** | 18 (45.0%) | 12 (40.0%) | 30 (42.9%) |
| **Procedure** | 27 (67.5%) | 10 (33.3%) | 37 (52.9%) |
| **Medication** | 31 (77.5%) | 28 (93.3%) | 59 (84.3%) |
