# Supplementary material for: Characterizing healthcare resource utilization in two rare diseases (Kleefstra syndrome and SLC6A1 epileptic encephalopathy) using multimodal real-world data
Source: Orphanet J Rare Dis. 2025 Jul 7;20:344. doi: 10.1186/s13023-025-03879-x (PMC12232648; doi:10.1186/s13023-025-03879-x)
Supplement: Supplementary file 3 — Additional file3 [file 13023_2025_3879_MOESM3_ESM.docx]

**Additional File 3. Demographics of individuals participating in survey-based portion of the study.** KS = Kleefstra syndrome.

|  | **KS**  **(N=6)** | **SLC6A1**  **(N=19)** | **Total**  **(N=25)** |
| --- | --- | --- | --- |
| **Age** |  |  |  |
| Mean (SD) | 7.33 (8.57) | 9.47 (7.49) | 8.96 (7.63) |
| Median [Min, Max] | 3.50 [2.00, 24.0] | 6.00 [2.00, 26.0] | 5.00 [2.00, 26.0] |
| **Biological sex** |  |  |  |
| Female | 2 (33.3%) | 13 (68.4%) | 15 (60.0%) |
| Male | 4 (66.7%) | 6 (31.6%) | 10 (40.0%) |
| **Race** |  |  |  |
| More than one race | 1 (16.7%) | 1 (5.3%) | 2 (8.0%) |
| White | 4 (66.7%) | 17 (89.5%) | 21 (84.0%) |
| Unknown | 1 (16.7%) | 1 (5.3%) | 2 (8.0%) |
| **Ethnicity** |  |  |  |
| Non-Hispanic | 5 (83.3%) | 16 (84.2%) | 21 (84.0%) |
| Hispanic | 0 (0%) | 2 (10.5%) | 2 (8.0%) |
| Unknown | 1 (16.7%) | 1 (5.3%) | 2 (8.0%) |
