# Supplementary material for: Characterizing healthcare resource utilization in two rare diseases (Kleefstra syndrome and SLC6A1 epileptic encephalopathy) using multimodal real-world data
Source: Orphanet J Rare Dis. 2025 Jul 7;20:344. doi: 10.1186/s13023-025-03879-x (PMC12232648; doi:10.1186/s13023-025-03879-x)
Supplement: Supplementary file 4 — Additional file4 [file 13023_2025_3879_MOESM4_ESM.docx]

**Additional File 3. Impact of diagnosis survey: respondent quotes by theme.** Representative quotes have been edited for spelling, and potentially identifiable information has been redacted or altered to protect participant privacy (e.g., participant name, participant gender, health insurance provider name). KS = Kleefstra syndrome.

| **Theme** | **Quote #** | **Representative Quotes Supporting Theme** |
| --- | --- | --- |
| Medical care changes | 1.1 | “After [the participant’s] diagnosis and researching the information available on Kleefstra syndrome, found that olanzapine has been [e]ffective and [their] medication was changed from Seroquel to olanzapine.” (KS) |
| Access to care | 2.1 | “Doctors began believing that I needed the resources I kept asking to receive for my [child].” (SLC6A1) |
| Insurance | 3.1 | “We were able to qualify for state Medicaid and increase therapies by double.” (SLC6A1) |
|  | 3.2 | “Developmental therapies are not covered by our insurance.” (SLC6A1) |
|  | 3.3 | “It's really a shame such a diagnosis has yielded no additional support or benefit from the healthcare community. Both [a private insurance company] and [public health insurance] have denied us services we need.” (SLC6A1) |
| Value | 4.1 | “The impact of receiving a Kleefstra syndrome diagnosis has been tremendous for the family. Just knowing what [the participant] has, understanding [their] diagnosis and mostly, having access to other parents and their experiences via online Kleefstra syndrome groups has changed our lives and ability to cope.” (KS) |
|  | 4.2 | “Simply knowing what [the participant’s] diagnosis was provided direction and a wealth of information that would not have been looked at without the diagnosis.” (KS) |
|  | 4.3 | “Before [the participant] was diagnosed, we thought it was something [they] would outgrow. Neurologist would tell us a lot of kids outgrow epilepsy. Once we received the actual diagnosis, we [k]new it was something that [the participant] would not outgrow and had to take more action in getting [them] the right care, more therapies, and we even had to switch… schools. [The participant] now goes to a school for kids with learning disabilities.” (SLC6A1) |
| Validation | 5.1 | “Diagnosis [as a young adult] for my [child] was more of a validation as [their] educational & medical supports have been in place for many years.” (SLC6A1) |
| No impact | 6.1 | “There is no one who can help. We go to all these specialist[s] and get no help. Constant road blocks. Have no idea w[h]ere life will lead.” (SLC6A1) |
